# Chemical and Biological Aspects of Montanine-Type Alkaloids Isolated from Plants of the Amaryllidaceae Family

**DOI:** 10.3390/molecules25102337

**Published:** 2020-05-16

**Authors:** Darja Koutová, Negar Maafi, Radim Havelek, Lubomír Opletal, Gerald Blunden, Martina Řezáčová, Lucie Cahlíková

**Affiliations:** 1 Department of Medical Biochemistry, Faculty of Medicine in Hradec Králové, Charles University, Šimkova 870, 500 03 Hradec Králové, Czech Republic; koutova.darja@lfhk.cuni.cz (D.K.); havelekr@lfhk.cuni.cz (R.H.); rezacovam@lfhk.cuni.cz (M.Ř.); 2 ADINACO Research Group, Department of Pharmaceutical Botany, Faculty of Pharmacy, Charles University, Heyrovského 1203, 500 05 Hradec Králové, Czech Republic; negarm@faf.cuni.cz (N.M.); opletal@faf.cuni.cz (L.O.); 3 School of Pharmacy and Biomedical Sciences, University of Portsmouth, Portsmouth, Hampshire P01 2DT, UK; gblunden10@gmail.com

**Keywords:** alkaloids, Amaryllidaceae, biological activity, derivatives, montanine, montanine-type, pancracine

## Abstract

Plants of the Amaryllidaceae family are promising therapeutic tools for human diseases and have been used as alternative medicines. The specific secondary metabolites of this plant family, called Amaryllidaceae alkaloids (AA), have attracted considerable attention due to their interesting pharmacological activities. One of them, galantamine, is already used in the therapy of Alzheimer’s disease as a long acting, selective, reversible inhibitor of acetylcholinesterase. One group of AA is the montanine-type, such as montanine, pancracine and others, which share a 5,11-methanomorphanthridine core. So far, only 14 montanine-type alkaloids have been isolated. Compared with other structural-types of AA, montanine-type alkaloids are predominantly present in plants in low concentrations, but some of them display promising biological properties, especially in vitro cytotoxic activity against different cancerous cell lines. The present review aims to summarize comprehensively the research that has been published on the Amaryllidaceae alkaloids of montanine-type.

## 1. Introduction

Amaryllidaceae is a family of monocotyledonous plants that are widely distributed over the tropical and warm regions of the world, especially in the southern African region. Species of some genera are also found in the Mediterranean area and temperate regions of Asia [1,2]. The Amaryllidaceae is one of the 20 most important alkaloid containing plant families. Up to now, more than 600 structurally diverse alkaloids have been isolated from plants of this family with a wide range of interesting biological properties, including antitumor, antifungal, antibacterial, antiviral, antimalarial, analgesic, and antineurodegenerative activities [3,4,5,6].

Plants of the family Amaryllidaceae have a long history of usage as herbal remedies all over the world to cure different ailments and diseases [3,7,8,9,10]. For example, *Hymenocallis litorallis* has been used as a centuries-old remedy for cancer in the traditional medicine of the Mayan people of South America [9]. A decoction of leaves of *Zephyranthes candida* has been utilized in South America as a remedy for diabetes mellitus [10], and the bulbs of *Boophone disticha* in southern Africa for cancer remediation by the indigenous Sotho, Xhosa and Zulu people [3,11]. The medicinal properties of these plants were already known in the fourth century B.C., when Hippocrates of Cos used oil from the daffodil *Narcissus poeticus* to treat uterine tumors [12,13]. Interestingly, this plant was also described in the Bible as a treatment of symptoms related to cancer [13]. 

Since the isolation of the first alkaloid, lycorine, from *N. pseudonarcissus* in 1877, Amaryllidaceae alkaloids (AA) have been attractive sources for chemical investigations, and many of them have been isolated, screened for different biological activities, and synthesized by a number of research groups. The most-known Amaryllidaceae alkaloid is galantamine, which is used in the form of its hydrobromide salt for the treatment of mild and severe stages of Alzheimer’s disease [14].

## 2. Biosynthesis, Phytochemistry and Occurrence of Montanine-Type Alkaloids

**Figure 1:**
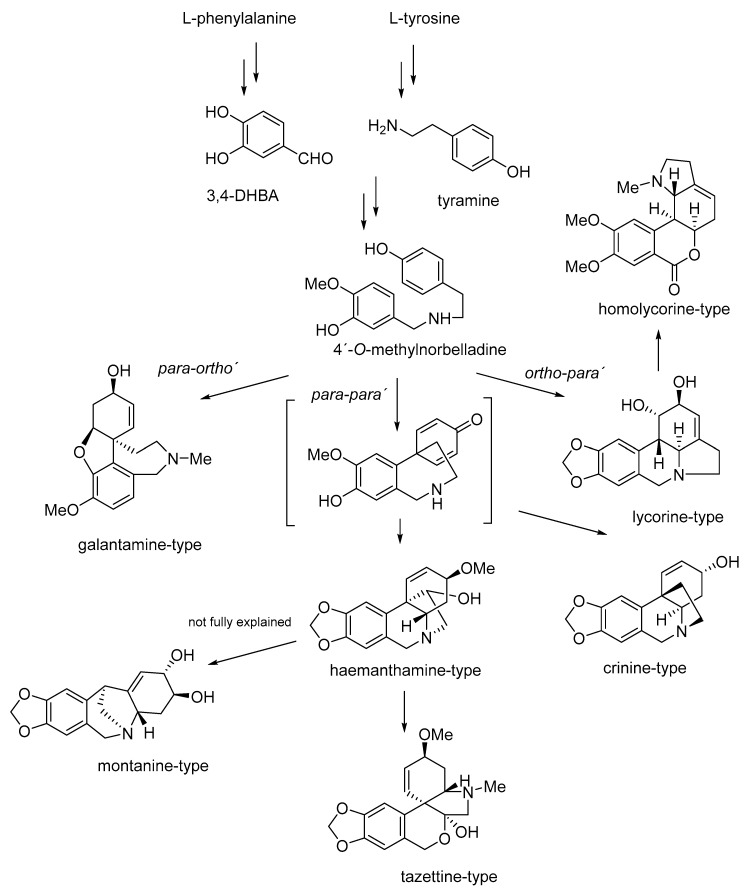
Biosynthetic pathway of main structural types of Amaryllidaceae alkaloids.

Amaryllidaceae alkaloids are synthesized within the norbelladine pathway from the aromatic amino acids phenylalanine and tyrosine, which are used to produce key intermediates in the biosynthesis of 4′-*O*-methylnorbelladine [15]. This key intermediate is formed through the condensation of 3,4-dihydroxybenzaldehyde (also known as protocatechuic acid; 3,4-DHBA) produced from L-phenylalanine and tyramine formed from L-tyrosine (Figure 1). As the result of condensation of intermediates, a Schiff base is generated, which is reduced to norbelladine. The biosynthesis continues with methylation of norbelladine by 4′-*O*-methyltransferase to 4′-*O*-methylnorbelladine, as a central intermediate for the biosynthesis of most AA (Figure 1) [15,16,17]. This key intermediate undergoes three different types of intramolecular oxidative couplings: *ortho-para*′, *para-para*′, and *para-ortho′*, which lead to the formation of the basic structural types of AA: norbelladine, lycorine, homolycorine, galantamine, haemanthamine, crinine, narciclassine, tazettine and montanine (Figure 1). Generally, genes involved in the biosynthesis of AA are poorly studied and transcriptomic studies, as well as genome sequencing, were only recently initiated for this plant family [16,18,19,20]. 

**Figure 2:**
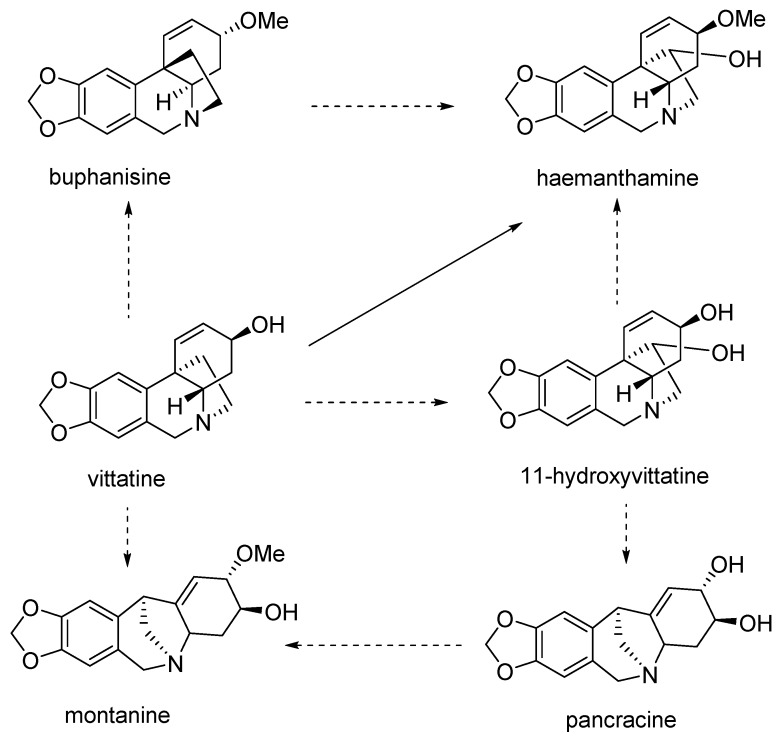
Proposed biosynthesis of montanine and pancracine, according to Feinstein and Wildman [23].

Studies dealing with the biosynthesis of montanine type alkaloids are more or less controversial [21,22]. In the study reported in 1976, 11-hydroxyvittatine was suggested as the main precursor for montanine and haemanthamine biosynthesis in *Rhodophiala bifida*, mentioning the higher conversion ratio for haemanthamine in comparison with montanine caused by skipping the rearrangement step in the biogeneration of haemanthamine from 11-hydroxyvittatine. In this study, the rearrangement of 11-hydroxyvittatine to pancracine, and then 2-*O*-methylation of pancracine, was described as a possible pathway for the biosynthesis of montanine in this plant [23]. The proposed biosynthesis of montanine, and relationships within some AA of the crinine- haemanthamine and montanine-type described by Feinstein and Wildman, are summarized in Figure 2. A similar biosynthetic pathway for montanine-type alkaloids has been described, with slight modifications, in further studies [16,24].

**Figure 3:**
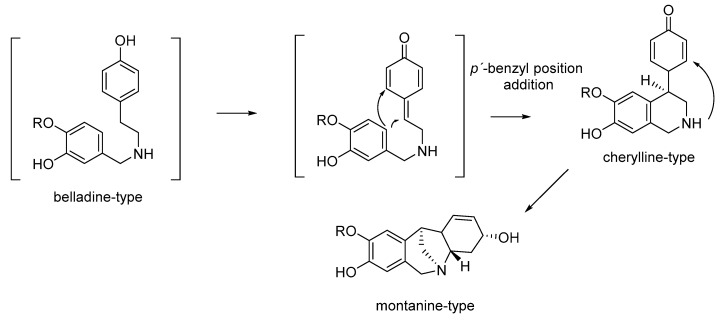
Proposed biosynthetic pathway for montanine-type, according to Jin [25].

Another biosynthetic pathway for montanine-type AA was described by Jin in 2007, who proposed the primary biosynthetic pathways of each type of AA [25]. These supposed the formation of cherylline-type AA by intramolecular addition to the *p′*-position of the electronic-rich aromatic ring to the benzylic position of the oxidized quinonoid form. Further addition of the secondary amine to the intermediate dienone provides montanine-type AA (Figure 3).

**Figure 4:**
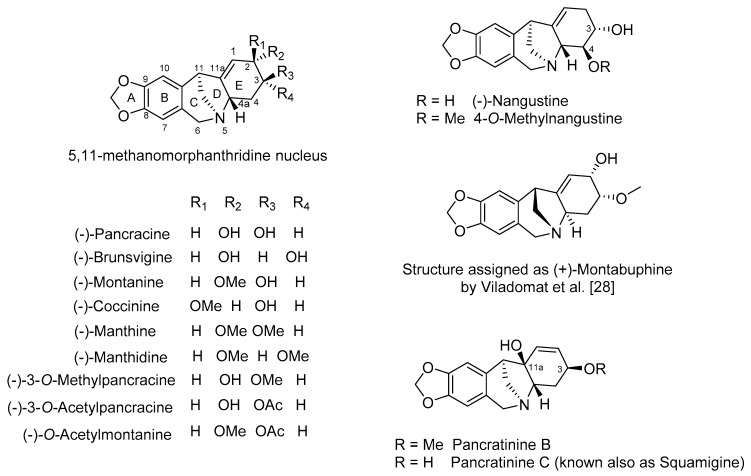
Chemical structures of montanine-type Amaryllidaceae alkaloids.

The first AA of montanine-type were isolated by Wildman et al. in 1955 from a *Haemanthus* species [26], namely coccinine, manthidine, manthine and montanine (Figure 4). Further congeners were identified and isolated from plants in the following decades (Table 1). Up to now, fourteen AA possessing an intriguing pentacyclic 5,11-*b*-methanomorphanthridine ring system as a core skeleton have been isolated from different Amaryllidaceae species (Figure 4, Table 1). According to the different position of the C=C double bond in the E ring of the alkaloid skeletons, they can be divided into two subgroups (Figure 4). Alkaloids from pancracine to montabuphine belonging to one of the representative subgroups possess the double bond between C1 and C11a. Most alkaloids within this subgroup differ from each other only in the configurations and oxygen-containing substitutions on stereocenters at C2 and C3 in ring E (Figure 4). Nangustine and 4-*O*-methylnangustine display different substituents in positions C3 and C4. Pancratinine B and pancratinine C (also known as squamigine), both isolated from *Pancratium canariense* [27], are characterized by the presence of a double bond between C1 and C2 and with a hydroxy group at C11a (Figure 4). In 1995, Viladomat et al. reported the isolation of montabuphine from bulbs of *B. flava*, describing the β-orientation for the methano-bridge for the first time [28]. This isolation has attracted attention, because this suggested that both enantiomeric forms of the montanine-type framework occur in nature. More recently, obtained data from the total synthesis of (+)-montabuphine found the previous spectroscopic published data to be controversial for the presented structure, and its legitimate structure is yet to be revised [29,30]. The overview of plants from which montanine-type AA have been isolated is summarized in Table 1.

## 3. Biological Activity of Montanine-Type Amaryllidaceae Alkaloids

Many studies have shown the antiproliferative, cytotoxic, antifungal, antibacterial, antimalarial, and anticholinesterase effects of AA [54,55,56,57,58]. The galantamine-type alkaloid galantamine is already used in the therapy of AD. Lycorine, haemanthamine, pancratistatin and narciclasine were found to display significant antiproliferative, cytotoxic and apoptotic properties across multiple cancer cell lines in vitro or the suppression of tumor growth in animal models of cancer in vivo [55,59,60,61]. As in the case of the potent pharmacological activities of lycorine-, haemanthamine- and narciclasine-type AA, the biological activities of montanine-type AA have also been studied, and today these structures belong to the most intensely investigated, since they have been reported to possess potent growth-inhibitory effects against diverse cancer cell lines [33,43,62,63]. It has also been shown that some montanine-type AA display anxiolytic, antidepressive and anticonvulsive activities, as well as immunomodulatory properties [42]. Moreover, montanine itself has also been studied for its acetylcholinesterase inhibition, and anti-rheumatic, antibacterial and antifungal effects [31,64,65].

### 3.1. Anticancer Potential of Montanine-Type Amaryllidaceae Alkaloids

The first evidence for montanine-type AA as an interesting group of plant-derived compounds that display growth inhibition and cytotoxicity to cancer cells in vitro was reported for montanine, isolated from bulbs of *Hippeastrum vittatum* in 2008 [35]. Since then, there have been other studies dealing with a group of montanine-type AA and their effect on proliferation and viability of cancer cells. Cytotoxicities, expressed as 50% inhibitory concentration (IC_50_) values, for the antiproliferative activity of montanine-type AA in vitro against different cancer and non-cancer cell lines are summarized in Table 2. Either the IC_50_ or GI_50_ values quoted in the published works included in this table were determined using standard colorimetric assays, based on either the reduction of the tetrazolium salt WST-1 and MTT to formazan by mitochondrial dehydrogenases or an alternative quantitative assay based on the measurement of cellular protein content, using the protein-binding dye sulforhodamine B (SRB).

The previously mentioned work of Silva et al. [35] comparing montanine with vittatine reported the growth inhibitory activity of montanine against five human cell lines, colon adenocarcinoma HT29, non-small cell lung carcinoma H460, renal cell carcinoma RXF393, breast carcinoma MCF7 and epithelial ovarian cancer OVCAR3, with IC_50_ values in the low µM range [35]. According to the study of Al Shammari et al. [43], montanine strongly decreased the growth of 7 different adherent cancer cell lines of several histotypes by treatment with a single 10 µM dose. Montanine was also able to inhibit the cell growth of human leukemic cell lines MOLT-4 and Jurkat with a single dose of 10 µM, causing growth percentage GP values of 4% and 2%, respectively. All of these cell lines were tested for determination of IC_50_ values, using 48 hours’ treatment and WST-1 assay. As can be seen in Table 2, Al Shammari et al. [43] used colon adenocarcinoma HT29 and breast carcinoma MCF7 cell lines and, similar to the study of Silva et al. [35]; values were in the low µM range (in the study of Silva et al. [35], 0.71 µg/mL corresponds to 4.7 µM for HT-29, and 0.74 µg/mL to 4.9 µM for MCF-7), although the second mentioned study does not specify the time interval over which montanine had been applied to the determined cell lines. Not only these, but also other adherent cancer cell lines used in the study (pancreatic carcinoma PANC-1, ovarian carcinoma A2780, cervix carcinoma HeLa, osteosarcoma SAOS-2), were very strongly inhibited by montanine treatment in low µM concentration. The concentration producing 50% inhibition of proliferation of a cancer cells model resistant to apoptosis (lung adenocarcinoma A549) is even lower and was 1.09 ± 0.31 µM in this study [43]. Instead of these human adherent cancer cell lines, the IC_50_ values of leukemic Jurkat and MOLT-4 cell lines were very low, resulting in values of 1.04 ± 0.14 µM and 1.26 ± 0.11 µM. 

Another recent study of Masi et al. [33] confirmed the strong antiproliferative and cytotoxic effect of montanine on cancer cell death, using a panel of six cancer cell lines, including human breast carcinoma MCF-7, mammary gland Hs578T, adenocarcinoma MDA-MB-231, human colon carcinoma HCT-15, human lung carcinoma A549 and human melanoma SK-MEL-28 [33].

The study of Masi et al. [33] was also the first work reporting the cytotoxicity of coccinine, which was co-isolated with montanine from *Haemanthus humilis*. The antiproliferative effect of coccinine reported in this study revealed this montanine-type alkaloid as being the more promising anticancer agent. However, despite the promising observations on the activity of coccinine during the cell culture experiments, its overall cytotoxicity was less than that of montanine [33].

Another important montanine-type AA, pancracine, isolated from *N.* cv. Professor Einstein, displayed significant cytotoxic effects [62]. The first screening test for cytotoxicity revealed the ability of 10 µM pancracine treatment to reduce the viability of 9 cancer cell lines, including Jurkat, MOLT-4, A549, MCF-7, A2780, HT-29, PANC-1, HeLa and SAOS-2. Except for PANC-1, the IC_50_ values for all the remaining cell lines were determined; values ranged from 2.20 to 5.15 µM, as described in detail in Table 2 [62].

Antiproliferative activities were also achieved with pancracine isolated from *P. canariense* [47]. This structure-activity study was mentioned earlier in the section of derivatives of montanine type AA. Nevertheless, it is important to highlight the strong growth inhibitory effect of pancracine treatment observed in a mini-panel of human solid tumors derived from ovarian tissue (A2780), lung (SW1573), breast (T-47D) and colon (WiDr) [47]. As in the study of Breiterová et al. [66], A2780 had a similar IC_50_ value after 48 hours’ treatment.

In vitro growth-inhibitory effects of another montanine type AA, manthine, against cancer cells resistant to (A549, SK-MEL-28, U373) and sensitive to (MCF-7, Hs683, B16F10) apoptosis suggest that manthine is capable of overcoming apoptosis resistance [63]. In the same study, manthine also significantly reduced the proliferation of the GSC22 cancer cell line at a concentration as low as 1 µM and also inhibited proliferation by 95% at concentrations of 10 and 30 µM. Manthine was, surprisingly enough, more efficient than haemanthamine [63].

From the papers published so far, it appears that montanine, the main representative of montanine-type AA, is the most effective in reducing the growth of cancer cell lines within this group of structurally related compounds. On the other hand, the relevance of this conclusion is uncertain, since only four of these compounds have been tested for cytotoxicity so far. It is evident from the results described that further efforts should be made to reveal the mechanism of the cytotoxic effect of these substances and a detailed mode of action can lead to better subsequent in vivo testing. Since many different types of solid and leukemic cancer cell lines have been used to study cytotoxicity, it can be concluded that montanine type AA are promising agents in the field of therapy of human cancer diseases.

### 3.2. Other Biological Activities of Montanine-Type Amaryllidaceae Alkaloids

As already mentioned above, montanine-type alkaloids are not abundant in plants, thus only a few pilot studies have been reported on other biological activities of these compounds.

Montanine and pancracine were screened for their antibacterial activity in two different studies [65,67]. Montanine was the more active of the two alkaloids against pathogenic *Escherichia coli*, *Pseudomonas aeruginosa*, *Staphylococcus aureus* and *S. epidermis*, giving values of 5, 20, 5 and 15 µg respectively, as minimum quantities required for activity [67]. The antibacterial and antifungal activity of pancracine has been studied using an agar diffusion technique against a Gram-positive-bacterium *S. aureus*, two Gram-negative bacteria *E. coli* and *P. aeroginosea*, and *Candida albicans* [65]. Pancracine displayed activity against *S. aureus* and *P. aeroginosea* and moderate activity against *C. albicans*, with a MIC of 188 µg/mL. 

The in vitro antiparasitic activity of nangustine and pancracine isolated from *N. angustifolius* subsp. *transcarpathicus* against the protozoans *Trypanosoma brucei rhodesiense*, *T. cruzi*, *Leishmania donovani* and *P. falciparum* was reported in 2002 [46]. Pancracine showed a higher activity than nangustine against all four protozoan parasites tested (Table 3). While nangustine has been classified as inactive, pancracine showed weak activity against *T. brucei rhodesiense* and *T. cruzi*, but no activity against *L. donovani*. The IC_50_ values of 0.75 and 0.70 µg/mL, respectively, for two strains of *Plasmodium falciparum*, represented the weak antimalarial activity of pancracine. No cytotoxic activity was demonstrated for either alkaloid against L-6 cells (rat skeletal myoblasts) within this study [46].

Montanine has also been studied for its antiarthritic activity in antigen-induced and collagen-induced arthritis models [31]. The alkaloid significantly attenuated the development of experimental arthritis in both acute and chronic models [31], dose-dependently, with the lower dose being more effective in arthritis severity and paw nociception. This finding hypothesized that increased availability of montanine leads to an acute activation that culminates in mechanisms of desensitization of the receptor, reducing the activation of receptor cell signaling and, consequently, decreasing the biological effect seen at lower doses [31]. The obtained results indicated that montanine has a potential as a drug for autoimmune diseases, such as arthritis.

Two studies concerning the unique insight into biosynthesis regulation of AA montanine in the *R. bifida* plant [16] and its anti-rheumatic effect [31] are followed by a patent granted to the scientific group of prof. Zuanazzi in 2020 [68]. The embodiment of US20200000798A1 patent invention describes in detail the method of extraction of the alkaloid montanine, which is much faster than the previously described methods of isolation of alkaloids from *R. bifida* plant dispensing with numerous changes of solvent in order to strain the plant. The importance of this alkaloid is then evaluated by in vivo and in vitro tests as very promising in the treatment of anti-inflammatory diseases such as rheumatoid arthritis, ulcerative colitis, sepsis, acute pulmonary disease, inflammatory infections; in particular, inflammatory and fibrosing diseases related to the lungs and kidneys, osteoporosis. These drug candidate bioactivities were determined through biologically significant effect on the nociception, migration and proliferation of fibroblasts and lymphocytes and without changing or depressing the immune system.

AA are relevant options for the treatment of neurological disorders and neurodegenerative diseases. Montanine has been characterized behaviorally and toxicologically in a search for therapeutic applications by da Silva [42]. It has been found that montanine reduces locomotor activity and has sedative, anxiolytic, anticonvulsant and antidepressant effects in mice. Da Silva et al. suggested that montanine may act on the benzodiazepine site of the GABA receptor in mouse brain, and thus the anxiolytic, hypnotic effects of montanine could be caused by its combined action on several neurotransmitter receptor systems, including GABA receptors [42]. To the best of our knowledge, there is only one in silico study describing an interaction between montanine and γ-amino butyric acid type-A receptor associated protein (GABARAP), which is the ubiquitin-like modifier implicated in the intracellular trafficking of the GABA receptor [66] to study alkaloids with potential antiepileptic activity. On the other hand, a study revealing the mechanism of this action is still missing. Extracts of three *Haemanthus* species, *H. coccineus*, *H. montanus* and *H. sangiuneus*, and major alkaloid constituents, coccinine and montanine, were investigated in vitro for their affinity to the serotonin transporter protein SERT as potential antidepressants [34]. Both montanine and coccinine exhibited lower SERT affinity than estimated and did not explain the higher activity observed in the extracts.

Since galantamine, an AA, is used in the therapy of AD, AA and their semisynthetic derivatives have been intensively studied for their biological activity connected with the potential treatment of this disease [69,70,71,72]. So far, only two montanine-type alkaloids have been investigated for their potential to inhibit acetylcholinesterase. 4-*O*-Methylnangustine, isolated from *H. argentinum*, was not able to inhibit acetylcholinesterase activity significantly [36]. The effect of montanine on acetylcholinesterase activity has been studied at concentrations of 1, 500 and 100 µM. Montanine inhibited, in a dose-dependent manner, more than 50% of the enzyme at 1 mM concentration. With concentrations of 500 and 100 µM, 30–45% inhibition of AChE activity was detected [64]. The IC_50_ value was not determined within the study, but, from the obtained results, it can be deduced that the IC_50_ of montanine is higher than 100 µM. Compared with the IC_50_ value of galantamine (1.7 ± 0.1 µM [73]), montanine is recognized as a weak AChE inhibitor.

## 4. Preparation of Montanine-Type Alkaloids by Rearrangement of Haemanthamine-Type Ring System

**Figure 5:**
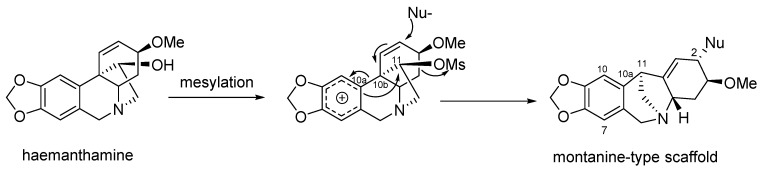
Tentative mechanism for haemanthamine-montanine skeletal rearrangement, according to Govindaraju et al. [63.

The unique structural features and promising biological activities associated with montanine-type AA and their low content in plant material have attracted considerable synthetic efforts [29,30,39,40,49,74,75,76,77,78,79,80,81,82,83,84,85,86,87,88,89,90,91]. However, most synthetic approaches involve many steps and low yield. For example, Hong et al., in 2008, reported the total synthesis of (−)-manthine involving over 18 steps [49]. Since multigram isolation of haemanthamine from plant material has been developed and old reports describe a semisynthetic transformation of the haemanthamine-type skeleton to the montanine-type scaffold [50,92], the preparation of montanine-type AA from the haemanthamine-type seems to be an elegant route for production of these alkaloids in higher amount compared with their total synthesis. The possibility of transformation of the haemanthamine ring type to the montanine-type skeleton through an intramolecular rearrangement was reported for the first time by Inubushi et al. in 1960 [50], within a study to define the absolute configuration of the newly discovered montanine-type alkaloids. During an experiment designed to find a method for replacing the C11 hydroxyl in crinine-type alkaloids with hydrogen, haemanthamine and crinamine were reacted with mesylchloride in pyridine and hydrolyzed by alkalized water. The result was the generation of two new compounds with a montanine-type scaffold, isomeric, but not identical to montanine and coccinine [50]. Manthine was also synthesized by the rearrangement of haemanthamine through mesylation and the addition of sodium methoxide as a nucleophile [50]. In 2009, Cedrón et al. [92] reported an unexpected rearrangement of the haemanthamine skeleton to a montanine skeleton in the presence of different halogenating agents like thionyl chloride (SOCl_2_), thionyl bromide and diethylaminosulfur trifluoride (DAST). Several experiments were performed to improve the yield. The different ratio of SOCl_2_ and the presence of base in the reaction mixture have been studied. When 1.5 equiv. of SOCl_2_ and 1.5 equiv. of pyridine were used, lower yields of products were obtained. The best results were achieved using a large excess of SOCl_2_. When 20 equiv. of SOCl_2_ were used, the yield improved to 71% of the montanine-type skeleton [92]. In 2018, a series of synthetic analogues of montanine-type alkaloids was reported by Govindaraju et al. [63] The mechanism of this rearangment has been described recently in detail, explaining the mesylated hydroxyl group at C-11 as a good leaving group being attacked intramolecularly by an electron rich aromatic moiety to create a new bond between C-10a and C-11, leading to the appearance of a 7-membered ring (Figure 5) [50,63].

## 5. Preparation and Structure Activity Relationship Studies on Synthetic Analogues of Montanine Type Alkaloids

The promising antiproliferative properties of some representatives of montanine-type AA, primarily of montanine and pancracine, initiated the development of synthetic analogues of these alkaloids. 

**Scheme 1:**
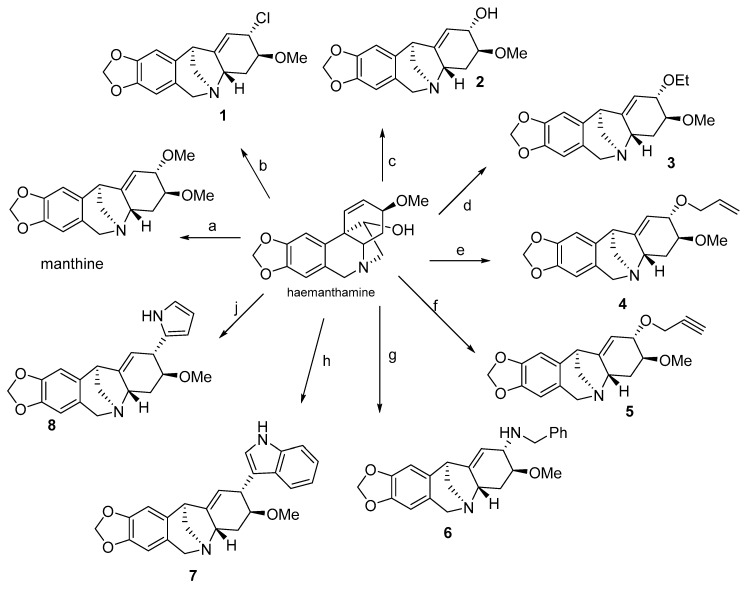
Preparation of C2-derived montanine-type derivatives, in order to study antiproliferative potential. Reagents and conditions for synthesis: (a) MsCl, py, 0 °C, 8 h, then MeOH, NaH (yield 65%); (b) MsCl, Et_3_N, CH_2_Cl_2_, rt, 48 h, (yield 55%) (c) MsCl, py, 0 °C, 8 h, then aq. NaHCO_3_ (yield 74%); (d) MsCl, py, 0 °C, 8 h, then EtOH, NaH (yield 65%); (e) MsCl, py, 0 °C, 8 h, then CH_2_=CH-CH_2_-OH, NaH (yield 68%) (f) MsCl, py, 0 °C, 8 h, then CH≡C-CH_2_-OH, NaH (yield 50%) (g) MsCl, py, 0 °C, 8 h, then BnNH_2_, NaH (yield 67%); (h) MsCl, py, 0 °C, 8 h, then indole, NaH (yield 62%); (j) MsCl, py, 0 °C, 8 h, then pyrrole, NaH (yield 55%).

**Scheme 2:**
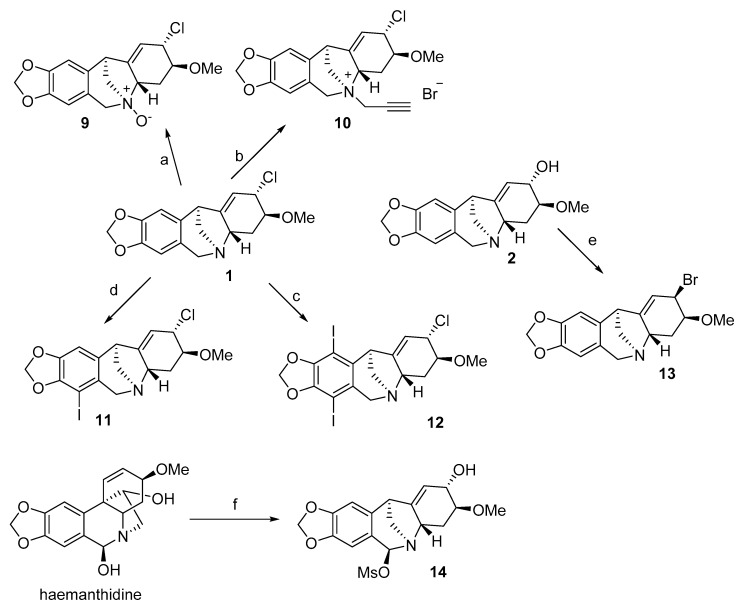
Further modifications of montanine derivatives and rearrangement of haemanthidine. Reagents and conditions for synthesis: (a) m-CPBA, CH_2_Cl_2_, rt, 30 min, (yield 70%); (b) MeCN, rt, CH≡C-CH_2_-Br, 24 h, (yield 78%) (c) NIS (2.4 eq), In(OTf)_3_, MeCN, 45 °C, 12 h, (yield 55%); (d) NIS, In(OTf)_3_, MeCN, 45 °C, 12 h, (yield 72%); (e) CBr_4_, Ph_3_P, CH_2_Cl_2_, rt, 4 h, (yield 64%) (f) MsCl (4 eq), py, 0 °C, 8 h, then aq. NaHCO_3_ (yield 46%).

A series of montanine derivatives has been developed under different reaction conditions (Scheme 1, Scheme 2). Various C2-substituted derivatives were prepared through the rearrangement of the haemanthamine ring by using different nucleophiles. Compounds **3**, **4** and **5** were prepared by using ethanol, allyl alcohol and propargyl alcohol respectively in tetrahydrofuran (THF) solution and treated with NaH. Compound **6** was prepared by substitution of the OH group at C2 using benzylamine. Compounds **7** and **8** were produced using indole and pyrrole dissolved in THF and alkalized with NaH. Compound **1** was formed through haemanthamine rearrangement using methansulfonyl chloride and then utilized in the synthesis of **9**, **10**, **11** and **12**, to investigate the possible effect of different substitutions in ring B and on nitrogen (Scheme 2). The detailed preparation of these derivatives can be found in Govindaraju et al. [63] Subsequently, montanine-type derivatives were screened for their antiproliferative activity on different cancer cell lines [63].

The antiproliferative properties of the synthesized compounds were evaluated in vitro using the MTT colorimetric assay on a panel of six cancer cell lines (A549, SKMEL-28, U373, MCF7, Hs683, B16F10); the results are summarized in Table 4. For the study of antiproliferative activity, the most explored position was C2, as the OH group is accessible for many chemical reactions. In the developed mini library, it has been demonstrated that the highest anticancer activity belongs to manthine, but a replacement of the methoxy group at position C2 by an ethoxy group, such as in 3, leads to reduction of antiproliferative activity, although ether substitutions of a three-membered carbon chain with multiple unsaturated bonds regain the activity. Although compound 7 showed interesting antiproliferative activity against different cell lines while containing a large indole substituent at position C2, overall, a small molecule replacement at C2, like OH, Cl and Br, is preferred for anticancer activity and the stereochemical configuration of C2 replacement does not seem critical for maintenance of activity, as demonstrated for derivatives 1, 2, and 13 (Table 4) [63].

Quaternization of the nitrogen produced inactive compounds like **9** and **10**, suggesting that charged derivatives cannot cross the cell membrane. Finally, the substitution pattern on ring B appears to be critical as derivatization at positions C7 and C10 in compounds **11** and **12** led to the loss of antiproliferative activity [63]. The rearrangement of haemanthidine to the corresponding montanine derivative **14** gave a completely inactive substance suggesting that C6 should be free to maintain antiproliferative activity (Scheme 2; Table 4) [47,63].

**Scheme 3:**
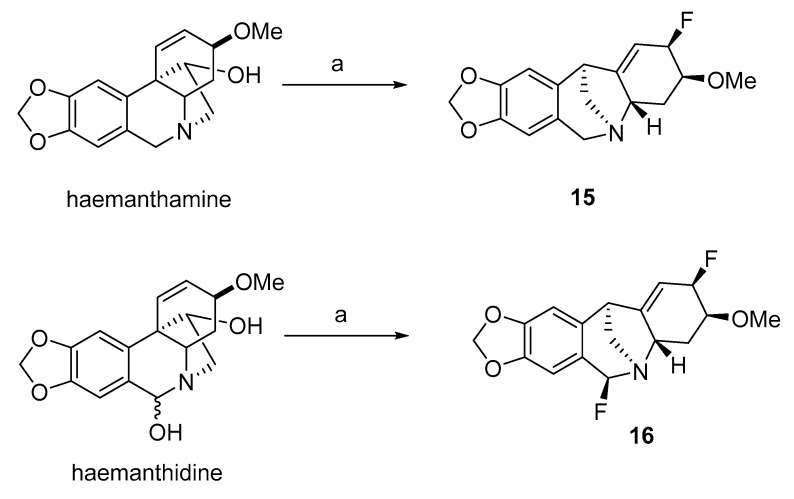
Preparation of further derivatives of montanine-type AA by rearrangement from haemanthamine and haemanthidine. Reagents and conditions for synthesis: (a) DAST, DCM, −78 °C, 24 h, (yield for **15** 60%; yield for **16** 49%).

Derivatives of montanine-type alkaloids **1**, **15**, and **16** developed by the rearrangement of haemanthamine and haemanthidine by Cedrón et al. in 2009 (Scheme 3) and pancracine were screened for their antimalarial potential against the F-32 Tanzanian strains of *P. falciparum* [92]. All the derivatives displayed higher activity (Table 5) than the natural alkaloid pancracine. The results indicated that the presence of one or more halogens is able to increase antimalarial activity [92].

## 6. Conclusions

This review summarizes the phytochemical and biological investigations of montanine-type AA. These alkaloids bear a characteristic 5,11-methanomorphanthridine structural core. To date, only 14 montanine-type AA have been isolated from nature, suggesting that they are quite rare. Some representatives of these alkaloids have been studied for different biological activities. Because of their unique architecture and promising antitumor activity, these alkaloids attracted synthetic effort for total synthesis, and also for the preparation of derivatives using several halogenating agents to study structure-activity relationships. Interestingly, montanine-type alkaloids can be easily obtained from alkaloids with a haemanthamine skeleton, which are more common and available from Amaryllidaceae plants. In the light of the presented overview of scientific data, the montanine-type AA can be recognized as an interesting source for the development of new drugs for the treatment of various diseases.

## Figures and Tables

**Table 1:** Reported isolation and identification of montanine-type alkaloids in Amaryllidaceae plants.

Alkaloid	Amaryllidaceae Family Plants	References	Ref. for Spectroscopic Data (NMR, MS, UV, IR)
Montanine	*Rhodophiala bifida*	[16,31,32]	[33,34,35,36,37,38,39,40]
	*Haemanthus humilis*	[33]	
	*Haemanthus amarylloides*	[26]	
	*Haemanthus coccineus*	[26,34]	
	*Haemanthus montanus*	[26,34]	
	*Haemanthus sanguineus*	[34]	
	*Haemanthus pauculifolius*	[41]	
	*Haemanthus deformis*	[41]	
	*Hippeastrum vittatum*	[35,42]	
	*Hippeastrum* cv. Ferrari	[43]	
	*Hippeastrum* cv. Double King	[43]	
	*Hippeastrum* cv. Pretty Nymph	[43]	
	*Hippeastrum* cv. Spartacus	[43]	
	*Hippeastrum argentinum*	[36]	
	*Scadoxus multiflorus*	[37]	
Pancracine	*Rhodophiala bifida*	[16,32]	[36,39,40,44,45,46]
	*Pancratium canariense*	[27,47]	
	*Pancratium maritimum*	[44,45]	
	*Narcissus angustifolius* subsp. *transcarpathicus*	[46]	
	*Lycoris radiata*	[48]	
	*Hippeastrum* cv. Ferrari	[43]	
	*Hippeastrum* cv.Double King	[43]	
	*Hippeastrum* cv. Pretty Nymph	[43]	
	*Hippeastrum argentinum*	[36]	
Coccinine	*Haemanthus humilis*	[33]	[33,34,38,39,40]
	*Haemanthus amarylloides*	[26]	
	*Haemanthus coccineus*	[26]	
	*Haemanthus montanus*	[34]	
	*Haemanthus sanguineus*	[34]	
	*Haemanthus deformis*	[41]	
Manthine	*Haemanthus amarylloides*	[26]	[32,49]
	*Haemanthus montanus*	[34]	
	*Haemanthus tigrinus*	[50]	
Manthidine	*Haemanthus coccineus*	[26]	[40]
	*Haemanthus pauculifolius*	[41]	
	*Haemanthus deformis*	[41]	
Brunsvigine	*Brunsvigia radulosa*	[50]	[40,49,51]
	*Brunsvigia cooperi*	[52]	
Pancratinine B	*Pancratium canariense*	[27]	[27]
Pancratinine C (reported also as Squamigine)	*Pancratium canariense*	[27]	[27,45]
	*Pancratium maritimum*	[45]	
	*Lycoris radiata*	[48]	
Nangustine	*Narcissus angustifolius* subsp. *transcarpathicus*	[46]	[46]
3-*O*-Methylpancracine (reported also as isohaemanthamine)	*Lycoris radiata*	[48]	[32,49]
3-*O*-Acetylpancracine	*Rhodophiala bifida*	[16]	[32]
4-*O*-Methylnangustine	*Hippeastrum argentinum*	[36]	[36]
Montabuphine	*Boophone flava*	[28]	[28,29,30]
*O*-Acetylmontanine	*Rhodophiala bifida*	[53]	[39]

**Table 2:** Impact of montanine-type Amaryllidaceae alkaloids (AA) on proliferation of cancer and non-cancer cells using in vitro assays. Results are expressed as a (IC_50_), or b (GI_50_) in micromolar (µM) units, unless otherwise stated.

Montanine Type Alkaloid	Cell Line	Method of Assay/Time of Treatment	Value and Type of Half-Maximal Inhibitory Concentration		References
Montanine	Jurkat	WST-1/48 h	1.04 ± 0.14	a	[43]
	MOLT-4	WST-1/48 h	1.26 ± 0.11	a	[43]
	A549	WST-1/48 h	1.09 ± 0.31	a	[43]
	HT-29	WST-1/48 h	1.35 ± 0.47	a	[43]
	PANC-1	WST-1/48 h	2.30 ± 0.45	a	[43]
	A2780	WST-1/48 h	1.67 ± 0.29	a	[43]
	HeLa	WST-1/48 h	1.99 ± 0.22	a	[43]
	MCF-7	WST-1/48 h	1.39 ± 0.21	a	[43]
	SAOS-2	WST-1/48 h	1.36 ± 0.49	a	[43]
	MRC-5	WST-1/48 h	1.79 ± 0.50	a	[43]
	A549	MTT/48 h	1.9 ± 0.4	a	[33]
	HCT-15	MTT/48 h	6.8 ± 0.5	a	[33]
	SK-MEL-28	MTT/48 h	23.2 ± 1.9	a	[33]
	MCF-7	MTT/48 h	4.4 ± 0.4	a	[33]
	MDA-MB-231	MTT/48 h	3.4 ± 0.9	a	[33]
	Hs578T	MTT/48 h	3.6 ± 1.7	a	[33]
	HT-29	SRB/not specified	0.71 ± 0.1 µg/mL	a	[35]
	H460	SRB/not specified	0.57 ± 0.57 µg/mL	a	[35]
	RXF393	SRB/not specified	0.65 ± 0.01 µg/mL	a	[35]
	MCF7	SRB/not specified	0.74 ± 0.02 µg/mL	a	[35]
	OVCAR3	SRB/not specified	0.84 ± 0.11 µg/mL	a	[35]
Pancracine	Jurkat	WST-1/48 h	5.07 ± 0.31	a	[62]
	MOLT-4	WST-1/48 h	2.71 ± 0.25	a	[62]
	A549	WST-1/48 h	2.29 ± 0.43	a	[62]
	HT-29	WST-1/48 h	2.60 ± 0.51	a	[62]
	A2780	WST-1/48 h	5.08 ± 0.43	a	[62]
	HeLa	WST-1/48 h	5.03 ± 0.36	a	[62]
	MCF-7	WST-1/48 h	2.68 ± 0.37	a	[62]
	SAOS-2	WST-1/48 h	2.20 ± 0.25	a	[62]
	MRC-5	WST-1/48 h	5.15 ± 0.34	a	[62]
	A2780	SRB/48 h	8.3 ± 0.5	b	[47]
	SW1573	SRB/48 h	4.3 ± 0.7	b	[47]
	T47-D	SRB/48 h	6.5 ± 2.5	b	[47]
	WiDr	SRB/48 h	9.1 ± 1.0	b	[47]
Coccinine	A549	MTT/48 h	5.9 ± 0.8	a	[33]
	HCT-15	MTT/48 h	16.8 ± 1.8	a	[33]
	SK-MEL-28	MTT/48 h	>50	a	[33]
	MCF-7	MTT/48 h	7.9 ± 0.9	a	[33]
	MDA-MB-231	MTT/48 h	13.8 ± 0.8	a	[33]
	Hs578T	MTT/48 h	5.3 ± 0.4	a	[33]
Manthine	A549	MTT/72 h	3	b	[63]
	SKMEL-28	MTT/72 h	4	b	[63]
	U373	MTT/72 h	5	b	[63]
	MCF7	MTT/72 h	4	b	[63]
	Hs683	MTT/72 h	3	b	[63]
	B16F10	MTT/72 h	3	b	[63]

**Table 3:** In vitro activity of nangustine and pancracine against parasitic protozoa [46].

Parasite	*Trypanosoma brucei rhodesiense*	*Trypanosoma cruzi*	*Leishmania donovani*	*Plasmodium falciparum*	
**Stage**	Trypomastigote	Trypomastigote	Amastigotes	Erythrocytic form	
**Strain**	STIB 900 IC_50_ (µg/mL)	Tulahuen C4 IC_50_ (µg/mL)	MHOM-ET-67/L82 IC_50_ (µg/mL)	K1 IC_50_ (µg/mL)	NF54 IC_50_ (µg/mL)
**Nangustine**	9.6	54.6	>30	2.14	2.93
**Pancracine**	0.7	7.1	>30	0.75	0.70

**Table 4:** Results of in vitro antiproliferative study of derivatives of montanine-type AA and manthine [63].

Compound	GI_50_ In Vitro Values (µM)					
	Resistant to Apoptosis			Sensitive to Apoptosis		
	A549	SKMEL-28	U373	MCF7	Hs683	B16F10
**1**	6	26	51	17	6	7
**2**	5	8	31	13	4	8
**Manthine**	3	4	5	4	3	3
**3**	59	>100	>100	82	>100	40
**4**	10	14	20	20	7	7
**5**	23	28	42	28	24	10
**6**	59	65	72	44	67	10
**7**	18	9	9	23	24	4
**8**	86	67	>100	68	95	11
**9**	>100	>100	>100	>100	>100	>100
**10**	>100	>100	>100	>100	>100	>100
**11**	>100	>100	>100	>100	>100	>100
**12**	78	>100	>100	78	71	39
**13**	9	18	25	19	5	7
**14**	>100	>100	>100	>100	>100	72

**Table 5:** Results of in vitro assay of derivatives **1**, **15**, **16** and pancracine against *Plasmodium falciparum* F-32.

Compound	IC_50_ (µg/mL)
Pancracine	0.9 ± 0.04
**1**	0.4 ± 0.02
**15**	0.6 ± 0.04
**16**	0.7 ± 0.04
Chloroquine	0.013
